# Probiotics and the Gut–Brain Axis: Emerging Therapeutic Strategies for Epilepsy and Depression Comorbidity

**DOI:** 10.3390/foods14172926

**Published:** 2025-08-22

**Authors:** Mustafa M. Shokr, Reem M. Eladawy, Yasmena O. Azar, Seham M. Al Raish

**Affiliations:** 1 Department of Pharmacology and Toxicology, Faculty of Pharmacy, Sinai University—Arish Branch, Arish 45511, Egypt; mostafa.mohsen@su.edu.eg (M.M.S.); reem.ali@su.edu.eg (R.M.E.); yasmena.osama@su.edu.eg (Y.O.A.); 2 Department of Biology, College of Science, United Arab Emirates University, Al Ain 15551, United Arab Emirates

**Keywords:** epilepsy, depression, probiotics, HPA axis

## Abstract

The bidirectional relationship between epilepsy and depression illustrates shared neurobiological mechanisms of neuroinflammation, hypothalamic–pituitary–adrenal axis dysregulation, and glutamatergic dysfunction. Depression is present in 20–55% of people with epilepsy, far greater than in the general population, while depression doubles epilepsy risk 2.5-fold, indicating shared pathophysiology. Neuroinflammatory mediators (interleukin-6, tumor necrosis factor alpha, high-mobility group box 1) establish a vicious cycle: seizures exacerbate inflammation and mood disruption, and stress lowers seizure thresholds. Hippocampal damage and cortisol toxicity also link these disorders, with early life stress imprinting lifelong risk via epigenetic alteration. Genetic studies identify pleiotropic genes (brain-derived neurotrophic factor) that regulate synaptic plasticity, serotonin activity, and immune responses. New treatments target shared pathways: ketamine and AMPAkines normalize glutamate tone; mGluR5 antagonists attenuate hyperexcitability and inflammation; DNA methyltransferase inhibitors reverse aberrant DNA methylation; and probiotics manipulate the gut–brain axis by boosting neuroprotective metabolites like butyrate. Despite challenges—transient effects, precision dosing, and blood–brain barrier penetration—these advances constitute a paradigm shift toward mechanistic repair rather than symptom management. The way forward includes clustered regularly interspaced short palindromic repeats (CRISPR)-based epigenome editing, biomarker-led therapies, and combination approaches (e.g., ketamine and probiotics). Such comorbidity needs to be managed holistically through integrated neuropsychiatry care, offering hope to patients with treatment-refractory symptoms.

## 1. Introduction

Epilepsy and depression have a strong bidirectional relationship, with the literature demonstrating depression in 20–55% of epilepsy patients, far more than the 5% prevalence in the general population [1]. On the other hand, depressed patients have a 2.5-fold increased risk of developing epilepsy, indicating shared neurobiological mechanisms beyond psychological factors [1,2]. This association appears in various patterns over time: depression precedes epilepsy in 30% of patients (suggesting common processes like neuroinflammation and stress system dysfunction), occurs after an epilepsy diagnosis in 50% (often due to seizure-related adversity and social stigma), or varies with seizure activity [2]. Sadly, depression in epileptic patients often goes unnoticed because it may present atypically as irritability or cognitive issues rather than classic symptoms, yet it carries a five-fold increase in suicide risk, especially among those with treatment-resistant epilepsy [3]. The outlook is particularly grim for drug-resistant epilepsy patients, who have a depression prevalence of 40–60%, nearly double that of patients with controlled seizures [4]. Several factors contribute to this increased vulnerability, including the psychological toll of uncontrolled seizures, neurological changes like limbic system dysfunction, side effects from medications due to polypharmacy, and structural brain abnormalities such as hippocampal sclerosis [5]. Chronic neuroinflammation and social challenges, including unemployment and stigma, further heighten the risk.

Compounding the problem, depression in drug-resistant epilepsy also goes unrecognized because symptoms like fatigue and cognitive impairment are common to epilepsy itself. This leads to poorer quality of life and increased mortality, highlighting the urgent need for better mental health screening [6,7]. Age is also a key factor in this comorbidity: children with epilepsy are three times more likely to develop depression than their peers and often show behavioral issues rather than typical depressive symptoms. Conversely, older patients tend to experience worse cognitive decline and seizure control if they are depressed, possibly due to neurodegenerative mechanisms, medication side effects, and social isolation [8,9]. Interestingly, this bidirectional relationship persists throughout life—early depression predicts later epilepsy, and vice versa—underscoring the importance of age-specific screening and integrated neurological and psychiatric care at all life stages [10,11].

## 2. Neuroinflammatory Pathways Linking Epilepsy and Depression: Different Mechanisms

### 2.1. Introduction to Neuroinflammation in Epilepsy–Depression Comorbidity

**Figure 1:**
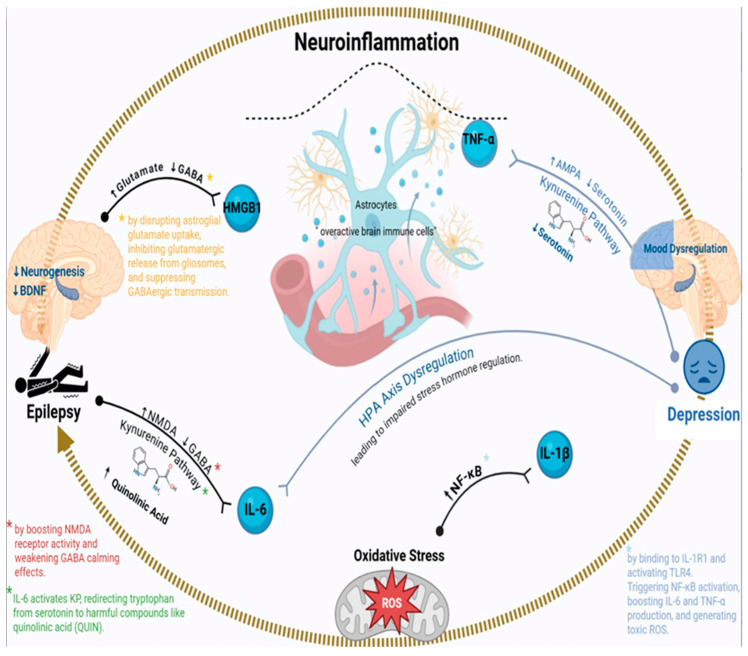
Mechanistic interplay of neuroinflammation in epilepsy and comorbid depression. This figure illustrates the central role of neuroinflammation in the two-way relationship between epilepsy and depression, emphasizing key molecular and cellular mechanisms. Neuroinflammation causes an imbalance in glutamate and GABA, with HMGB1 contributing to neuronal hyperexcitability. It also activates the Kynurenine Pathway, resulting in serotonin depletion and the production of neurotoxic metabolites like quinolinic acid, which lead to mood dysregulation and seizures. Pro-inflammatory cytokines (TNF-α, IL-6, IL-1β) sustain this inflammation. Oxidative stress, via ROS and NF-κB activation, further intensifies neuroinflammation. Chronic neuroinflammation also disrupts the HPA axis and lowers neurogenesis and BDNF levels, worsening emotional issues and seizure risk. This complex interaction underscores neuroinflammation as a key link in the co-occurrence of epilepsy and depression.

The strong connection between epilepsy and depression is now largely explained by shared neuroinflammatory processes, where abnormal immune activity fuels both increased brain excitability and mood issues [12]. Key inflammatory molecules (such as interleukin 6 (IL-6), tumor necrosis factor alpha (TNF-α), interleukin-1 beta (IL-1β), and high mobility group box 1 (HMGB1)) interfere with brain function by damaging synaptic connections, changing neurotransmitter levels, and disrupting stress response systems, which can trigger seizures while also worsening depressive symptoms [13]. These harmful cytokines are produced by overactive brain immune cells (microglia and astrocytes) and circulating immune cells, often in response to frequent seizures, prolonged stress, or a leaky blood–brain barrier [14]. This creates a destructive loop: epilepsy promotes inflammation and depression, while depression and inflammation, in turn, increase seizure risk, making the conditions mutually reinforcing (Figure 1).

### 2.2. Role of IL-6 in Epilepsy and Depression

IL-6, a multifunctional inflammatory molecule, shows increased levels in patients with both treatment-resistant epilepsy and major depression, where it promotes disease progression through several pathways [15]. In epilepsy, IL-6 makes brain cells more excitable by increasing N-methyl-D-aspartate (NMDA) receptor activity and weakening gamma-aminobutyric acid (GABA)’s calming effects. For depression, IL-6 overstimulates the body’s stress response system, the hypothalamic–pituitary–adrenal (HPA) axis, leading to impaired regulation of stress hormones and prolonged stress responses [16,17]. High IL-6 is linked to shrinkage of the hippocampus, a brain area often affected in both conditions, because it hinders new neuron growth and accelerates the loss of neural connections. Additionally, IL-6 activates the kynurenine pathway (KP), shifting tryptophan breakdown from serotonin production to generating harmful compounds like quinolinic acid (QUIN). This not only intensifies depressive symptoms but also increases brain susceptibility to seizures [17] (Figure 1).

### 2.3. TNF-α: A Dual Role in Neuronal Excitability and Mood Dysregulation

TNF-α has dual effects on brain cells; at low levels, it helps strengthen neural connections, but at high levels, it becomes harmful to neurons [18,19]. In epilepsy, TNF-α boosts brain activity by increasing α-amino-3-hydroxy-5-methyl-4-isoxazolepropionic acid (AMPA) receptors at synapses, which play a role in seizure development [20]. For depression, TNF-α interferes with serotonin and dopamine systems by altering how synapses function and reducing levels of brain-derived neurotrophic factor (BDNF), a protein essential for maintaining healthy neurons and stable mood. Another important effect is that TNF-α weakens the blood–brain barrier, allowing inflammatory substances from the body to enter the brain and prolong inflammation [21,22]. Notably, research shows that TNF-α-blocking drugs like etanercept can reduce both seizures and depression symptoms in animal studies, suggesting potential new treatment options [23,24] (Figure 1).

### 2.4. HMGB1-TLR4 Signaling: A Key Link Between Neuroinflammation and Hyperexcitability

The HMGB1-TLR4 pathway has emerged as a crucial link between neuroinflammation and neuronal hyperexcitability, with a profound impact that extends beyond seizures and long-term stress [25,26]. In response to various clinical situations, HMGB1, a highly conserved nuclear protein, is actively secreted by immune cells, such as macrophages, monocytes, and dendritic cells, as well as by non-immune parenchymal cells, including neurons and glial cells. It also passes from necrotic cells [27]. The redox state of HMGB1 is crucial in regulating its biological activity; for instance, its disulfide form is highly active in cytokine production and acts as an essential damage-associated molecular pattern (DAMP) that alerts the innate immune system to cellular stress [27]. The effects of extracellular HMGB1 secretion extend beyond TLR4 activation; it also activates other key receptors, including the Receptor for Advanced Glycation End Products (RAGE), which amplifies the pro-inflammatory response in astrocytes and microglia [25,27,28,29].This widespread receptor engagement orchestrates a complex downstream signaling network. To establish a feed-forward amplification loop that sustains neuroinflammation, HMGB1-TLR4/RAGE signaling not only activates the NF-κB and mitogen-activated protein kinase (MAPK) pathways but also induces nitric oxide synthase (iNOS) and other pro-inflammatory mediators [30]. Most importantly, this prolonged stimulation significantly weakens the BBB’s structure, increasing its permeability. This disruption allows inflammatory mediators and peripheral immune cells to enter the central nervous system, which promotes local neuroinflammation and directly increases neuronal hyperexcitability [30]. Although HMGB1 clearly impairs astroglial glutamate uptake and suppresses GABAergic transmission, it also directly triggers glutamatergic release from glial subcellular particles (gliosomes) but not from neuronal synaptosomes, suggesting a distinct role for HMGB1 in regulating glutamate homeostasis through glial mechanisms [31]. The impact on synaptic function is also more subtle than previously reported. Moreover, by activating sphingomyelinase and Src kinases, HMGB1 can cause phosphorylation of NMDA receptor subunits, including NMDA receptor subunit epsilon-2 (NR2B), directly increasing neuronal excitability and seizure susceptibility [32].

The HMGB1-TLR4/RAGE pathway has emerged as a promising therapeutic target for neurological and psychiatric disorders because of its key role in neuroinflammation and hyperexcitability [33,34]. HMGB1 antagonists and TLR4/RAGE inhibitors are two preclinical approaches to blocking this system that have demonstrated therapeutic benefits in models of traumatic brain injury, neuroinflammation, epilepsy, and cognitive decline [34]. For example, drugs like pirfenidone, which reduce HMGB1 and TLR4 levels as well as other pro-inflammatory cytokines, have shown excellent effects in seizure models [35]. They also appear to enhance cognitive function, decrease oxidative stress, and influence neurotransmitter activity [36]. Besides confirming the mechanistic importance of HMGB1, these increased serum and cerebrospinal fluid levels in patients with epilepsy and depression suggest its potential as a biomarker for disease severity, prognosis, and even as a measure to monitor the success of new anti-inflammatory therapies for these debilitating conditions [36].

### 2.5. Microglial and Astrocytic Dysfunction in Epilepsy–Depression Comorbidity

A complex and dynamic function in regulating the pathogenic landscape of both illnesses is played by microglial and astrocytic impairment in the comorbidity of epilepsy and depression, which extends beyond simple hyperactivation and withdrawal in a linear fashion [37]. The resident immune cells in the brain, known as microglia, are highly adaptable and change their phenotype (M1 pro-inflammatory, M2 anti-inflammatory/repair) depending on the situation [37]. While the first point rightly emphasizes their pro-inflammatory M1-like activation and the production of excess IL-1β and TNF-α, which increase neuronal hyperexcitability and seizure susceptibility, their involvement in depression is equally important [37]. Excessive microglia produce neurotoxic chemicals that cause neuroinflammation and depressive-like behavior by over-activating the shunt tryptophan from serotonin production into the kynurenine pathway (KP) [34,38]. Prolonged microglial activation, especially the M1 phenotype, can worsen depressive symptoms by inhibiting hippocampal neurogenesis, a process vital for mood regulation and cognition. This ongoing neuroinflammation in the hippocampus, a region essential for mood and memory, creates a vicious cycle that exacerbates melancholy and epilepsy [39]. Chronic microglial dysfunction can cause abnormal synaptic pruning and impaired neural circuit function. Microglia directly influence synaptic plasticity, neurogenesis, and the release of neurotrophic factors. This inflammatory vicious loop is fueled by the P2X purinoceptor 7 (P2X7)-NLR family pyrin domain containing 3 (NLRP3)-IL-1β inflammatory axis, which is bolstered by excess ATP and CX3CL1, leading to breakdowns in brain networks and the blood–brain barrier (BBB) [40].

The most common glial cells in the brain, astrocytes, are also important participants, and their inability to control chronic neuroinflammation has a major impact on neuron homeostasis and circuit integrity [40]. Reactive astrocytes actively contribute to excitotoxicity and network instability rather than simply reducing their ability to absorb glutamate and buffer potassium [41]. Under inflammatory conditions, they can release several pro-inflammatory mediators (such as lipocalin-2 (Lcn2), glial cell-derived neurotrophic factor (GDNF), complement activation product (C3), chemokine (C-C motif) ligand 2 (CCL2), and C-X-C motif chemokine ligand 10 (CXCL10)), which prolong microglial activation and create a vicious positive feedback loop [42,43]. The inability of astrocyte potassium channels, such as inward rectifier-type potassium channels (Kir4.1), to buffer extracellular potassium results in neuronal hyperexcitability and increased susceptibility to seizures [41]. This dysfunction has been observed in both epilepsy and depression. Moreover, the syncytial network may be compromised by astrocytic gap junction disconnection, further disrupting their ability to maintain ion and neurotransmitter homeostasis [20]. Swollen astrocytes’ withdrawal and decreased BDNF release directly compromise synaptic health and neuroplasticity, compromising mood stability and seizure control [44]. Furthermore, glymphatic system malfunction and elevated oxidative stress caused by astrocyte dysfunction might hinder waste removal and create a toxic neuroenvironment [45]. Inflammatory signals from microglia trigger astrocyte activation, reactive astrocytes support microglial persistence, and the resulting chronic neuroinflammation is a hallmark of epilepsy–depression comorbidity [13]. This ongoing interaction between reactive microglia and dysfunctional astrocytes is an intrinsic self-sustaining cycle [13]. This complex dynamic between these glial cells emphasizes the need for therapies that aim to break the cycle of neuroinflammation and improve outcomes in these debilitating comorbid conditions by treating individual cellular dysfunctions and re-establishing normal glia–glia and glia–neuron interactions.

### 2.6. The Kynurenine Pathway: Bridging Inflammation to Serotonin Depletion

About 95% of tryptophan breakdown occurs via the KP, which is the primary pathway for tryptophan catabolism [46]. Its complex network of metabolites has a significant impact on neurological, immunological, and endocrine systems, extending far beyond just disrupting serotonin production [47]. Although inflammation—especially through cytokines like TNF-α and IL-6—triggers indoleamine 2,3-dioxygenase (IDO), diverting tryptophan from serotonin synthesis, the subsequent cascade within the KP produces both neurotoxic and neuroprotective metabolites, maintaining a delicate balance for normal brain function. QUIN is a potent NMDA receptor agonist that contributes to neuronal damage and excitotoxicity in various neurological conditions [48]. However, 3-hydroxykynurenine (3-HK), part of the neurotoxic arm of the KP, generates reactive oxygen species (ROS), which cause severe oxidative stress and neuronal death [49]. Many neuropsychiatric and neurodegenerative diseases—including Alzheimer’s disease, Huntington’s disease, schizophrenia, and AIDS dementia complex—are linked to imbalances in KP metabolites and are affected by neurotoxic metabolites [50].

On the other hand, KP also produces kynurenic acid (KYNA), an endogenous neuroprotector that functions by negatively interacting with both α7 nicotinic acetylcholine receptors and NMDA receptors (glutamate and glycine sites) [51]. Along with its anticonvulsant and anti-ischemic properties, KYNA also regulates dopamine and glutamate release in the brain. Enzymes such as kynurenine 3-monooxygenase (KMO) and kynurenine aminotransferases (KATs) precisely control the balance between neurotoxic QUIN and neuroprotective KYNA, often known as the “neurotoxic” and “neuroprotective” limbs of the KP. KMO activity is typically increased in inflammatory diseases, which shifts the balance toward neurotoxic metabolites and worsens neuroinflammation and neuronal death [52].

KP dysregulation significantly contributes to more frequent or severe seizures in epilepsy. By increasing glutamatergic excitotoxicity and inhibiting GABAergic regulation, elevated levels of neurotoxic kynurenines may lower the seizure threshold. The KP enhances neuronal vulnerability by contributing to mitochondrial dysfunction, an established factor in epilepsy’s development [53]. Prolonged IDO activation and the resulting increase in neurotoxic KP metabolites drive systemic and neuroinflammation in depression, leading to impaired neuroplasticity, decreased hippocampal neurogenesis, and disrupted dopamine transmission, which manifest as classic depressive symptoms like motivational deficits and anhedonia [54]. The comorbidity of depression and inflammation is thought to be explained by this inflammation-mediated tryptophan metabolism pathway [54].

Its direct involvement in immune-related disorders is evidenced by the complex regulation of KP enzymes by inflammatory mediators [55]. Investigating this pathway has revealed promising therapeutic possibilities for various mental and neurological conditions. The ratio of neurotoxic and neuroprotective metabolites can be restored by modulating the KP, such as blocking key enzymes like IDO or KMO, or by enhancing the production of neuroprotective KYNA [56]. Furthermore, several KP metabolites, such as kynurenine and 3-HK, have a connection between peripheral and central levels, indicating they could serve as diagnostic and prognostic markers of disease severity and treatment response in neurological and psychiatric disorders [57].

### 2.7. Oxidative Stress and Mitochondrial Dysfunction

Brain inflammation triggers the production of toxic reactive molecules (ROS/responsive neurostimulation) that destroy cells, damaging their membranes, proteins, and even genes, leaving the energy-producing mitochondria impaired [58,59]. In epilepsy, this mitochondrial damage depletes brain cells of energy and renders them more excitable and prone to seizures. In depression, the energy shortage disrupts the synthesis of mood-regulating neurotransmitters [60,61]. Notably, antioxidant treatments like N-acetylcysteine and resveratrol aim to relieve both by stripping away these pathogenic molecules and restoring the brain’s natural balance, offering double-barreled therapeutic benefits [60,61] (Figure 1).

## 3. HPA Axis Dysregulation in Epilepsy–Depression Comorbidity: Mechanisms and Therapeutic Implications

### 3.1. Introduction to HPA Axis Dysfunction in Neurological Disorders

**Figure 2:**
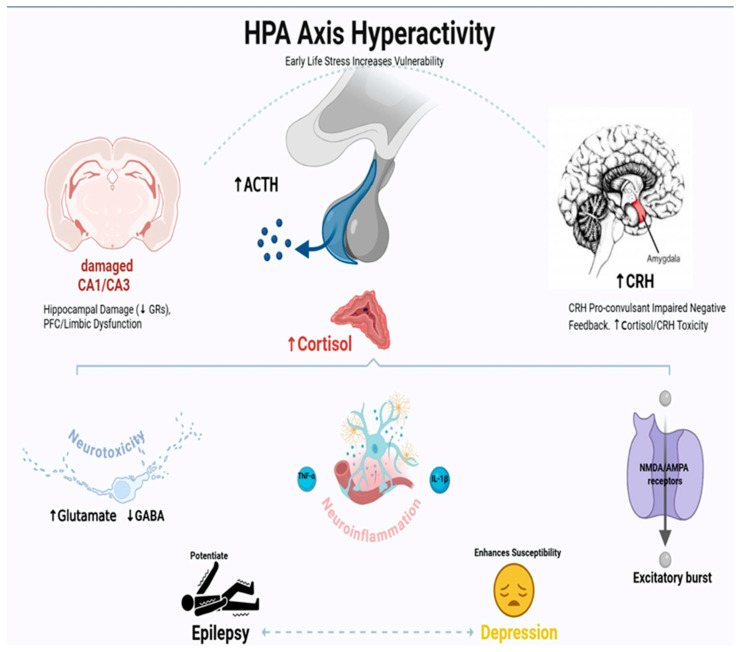
Role of HPA axis hyperactivity in the pathogenesis of epilepsy and depression.

The HPA axis is the primary system responsible for the body’s stress response, and its dysregulation has become crucial for understanding the link between epilepsy and depression [62,63]. Chronic activation of the neuroendocrine pathway creates a vicious cycle where epilepsy promotes HPA axis hyperactivity, which then increases the susceptibility to seizures and depression [62,63]. This two-way interaction is especially clear in TLE, where hippocampal damage disrupts the negative feedback mechanisms that normally regulate stress hormone release [62,63]. This secondary hypercortisolemia further worsens neuronal excitability, neuroinflammation, and mood dysregulation through several interconnected pathways, which we will explain in more detail [63,64] (Figure 2).

HPA axis hyperactivity is characterized by increased release of ACTH from the pituitary and, subsequently, elevated cortisol from the adrenal glands. This dysregulation is often caused by factors such as damage to the CA1/CA3 hippocampal regions, which impair negative feedback, and an overstimulated amygdala, leading to increased CRH release. The ongoing hyperactivity of the HPA axis promotes neuroinflammation (shown by activated glial cells), which is a key connection. Neuroinflammation contributes to neurotoxicity, the release of pro-inflammatory cytokines like TNF-α and IL-1β, and increased excitability through NMDA/AMPA receptors, causing excitatory bursts.

### 3.2. Neuroanatomical Basis of HPA Dysregulation in Epilepsy

The hippocampus and amygdala are only two of the neuroanatomical substrates involved in HPA axis dysregulation in epilepsy; a broader network of interconnected brain structures also participates, working together to influence stress responses and seizure susceptibility [65]. Other structures are vital in coordinating this dysfunction, even though hippocampal pathology—specifically neuronal loss in CA1/CA3 subfields and reduced glucocorticoid receptor (GR) density—unquestionably impairs the brain’s ability to inhibit stress responses and leads to chronic hyperarousal [66]. Corticotropin-releasing hormone (CRH) is secreted by neurosecretory neurons of the hypothalamic paraventricular nucleus (PVN), which serves as the primary central activator of HPA axis stress responses [67]. This core drive is generated by complex multisynaptic limbic forebrain circuits, which relay through subcortical regions rather than directly innervating the PVN [68].

The HPA axis is heavily suppressed by the prefrontal cortex, especially its medial part [69]. The prefrontal cortex’s top-down control of the HPA axis can be disrupted by trauma or dysfunction, which is common in epilepsy, leading to excessive and prolonged stress responses [65]. The HPA axis’s tone is also influenced by the bed nucleus of the stria terminalis (BNST), a critical limbic system relay that receives inputs from the hippocampus and amygdala before projecting to the PVN [67]. When these circuits are out of balance, CRH is released continuously, potentially causing hypercortisolemia. At the molecular level, the usual negative feedback loop that inhibits cortisol release becomes ineffective due to the breakdown of GRs in the hippocampus [70]. This results in persistent over-secretion of CRH, ACTH, and cortisol, chronically keeping the body in a stress state [70].

Both emotional regulation and seizure susceptibility are significantly affected by the vicious loop that is produced by the persistent activation of the HPA axis [71]. Chronic hyperglucocorticoids can worsen neuropathology and hasten epileptogenesis beyond their immediate pro-convulsant effects on neurons. Research indicates that activation of the HPA axis during seizures results from a disruption in the chloride gradient in CRH neurons, which is essential for effective GABAergic inhibition [63]. This disruption is caused by changes in the expression of K+/Cl− co-transporters such as potassium chloride cotransporter 2 (KCC2). Moreover, this disinhibits the HPA axis, increasing the risk of future seizures. Patients with epilepsy often experience high rates of anxiety and depression due to the chronic stress response, which not only affects neurogenesis and synaptic plasticity in various brain regions but also overstimulates the amygdala, making it more sensitive to stress and promoting CRH production [72]. Targeting the HPA axis—such as by blocking glucocorticoid effects with agents like RU486 or inhibiting CRH signaling with antagonists like antalarmin—is thus being explored as a strategy to improve seizure control while also addressing stress-related psychiatric comorbidities in epilepsy [72].

### 3.3. Glucocorticoid-Mediated Neurotoxicity and Seizure Threshold

Chronic hypercortisolism triggers a series of pathological effects on brain function that promote both depression and the development of epilepsy [73]. The stress hormone enhances neuronal excitability through two main mechanisms: increased glutamate levels alongside inhibition of the brain’s GABA system by reducing glutamic acid decarboxylase (GAD) enzyme activity [73,74]. These processes are mediated by both slow genetic mechanisms (via GR/MR receptors) and immediate, rapid mechanisms. Over time, cortisol overload also causes oxidative damage and reduces cellular energy production, making neurons more vulnerable to overactivation [75,76]. The hippocampus is particularly vulnerable to cortisol toxicity, as stress damages this region; it also disrupts the body’s stress response system, creating a self-perpetuating cycle where neurological and mood disorders reinforce each other [74,76] (Figure 2).

### 3.4. CRH and Its Role in Seizure Susceptibility and Mood Regulation

The CRH, the master regulator of the stress response system, directly contributes to seizure induction and depressive behaviors [76,77]. Besides its role in regulating hormone secretion, CRH also acts as a highly effective signaling molecule in brain areas like the amygdala and hippocampus. In these regions, CRH promotes excitatory signaling by increasing NMDA and AMPA receptor activity through its corticotropin-releasing hormone receptor 1 (CRHR1) pathway, while simultaneously suppressing the brain’s inhibitory mechanisms [78,79]. Animal studies confirm CRH’s pro-convulsant role when injected into the hippocampus, and medications that block CRHR1 are being developed as promising therapies for epilepsy and depression [80]. Interestingly, patients with treatment-resistant forms of both diseases show abnormally high CRH levels in their spinal fluid, implicating this stress molecule as a compelling target for new medications that could potentially treat both disorders simultaneously [80,81] (Figure 2).

### 3.5. HPA–Immune System Interactions: The Neuroinflammatory Link

The stress response system (HPA axis) and the immune system are in constant interaction, and this dialogue is disrupted in depression and epilepsy [71]. Repeated stimulation of the HPA axis causes resistance to cortisol’s anti-inflammatory effects, creating a self-perpetuating cycle where inflammation remains unchecked. This leads to increased levels of inflammatory cytokines (like IL-1β, IL-6, and TNF-α), which then impair the regulation of stress hormones [82,83]. In epilepsy, such an inflammatory state conditions the brain for seizures by disrupting the BBB, hyper-activating immune cells in the brain, and damaging vital support cells called astrocytes [82,83]. One of the key participants in this process is the KP—when activated by inflammation, it diverts tryptophan (which is normally used to produce serotonin) toward the creation of compounds that influence both seizure susceptibility and mood, forming a biological link between the two diseases [83,84] Figure 2.

### 3.6. Developmental Aspects: Early Life Stress and Vulnerability

Early life stress significantly raises the risk of depression and epilepsy by causing long-lasting changes in stress response systems [85]. Animal studies demonstrate that stressors like maternal separation lead to prolonged HPA axis hyperactivity, decrease stress-regulating receptors (GR) in the hippocampus, and increase CRH expression in brain areas involved in emotion processing [86]. These alterations establish lifelong patterns of hyperarousal to stress and increased seizure susceptibility. Human research supports this connection, showing that childhood trauma is associated with an earlier onset of epilepsy, more severe seizures, and greater susceptibility to comorbid depression [86,87]. These long-term effects originate from epigenetic mechanisms—specifically, chemical modifications of DNA, such as methylation of genes that regulate GR and CRH, which can permanently alter the brain’s responses to stress and emotional regulation [87,88].

### 3.7. Clinical Evidence and Biomarker Correlations

Clinical studies consistently show HPA axis dysfunction in depressed and epileptic patients [71,73]. Common findings include elevated basal cortisol secretion, reduced responses to dexamethasone suppression tests, and flattened diurnal cortisol patterns [73]. This dysfunction is linked to specific clinical signs: morning cortisol levels can predict seizure frequency in TLE patients, while evening cortisol levels relate to depression severity [71,73]. Magnetic resonance imaging reveals that hippocampal volume reduction closely associates with the degree of HPA regulation disruption and mood symptom severity [89]. Importantly, HPA abnormalities are often observed before depression develops in epilepsy patients, indicating a potential causal role rather than a secondary effect of chronic disease [65,89].

## 4. Genetic Predisposition as a Link Between Epilepsy and Depression: Shared Pathways and Mechanisms

### 4.1. Introduction to Genetic Overlap in Epilepsy and Depression

**Figure 3:**
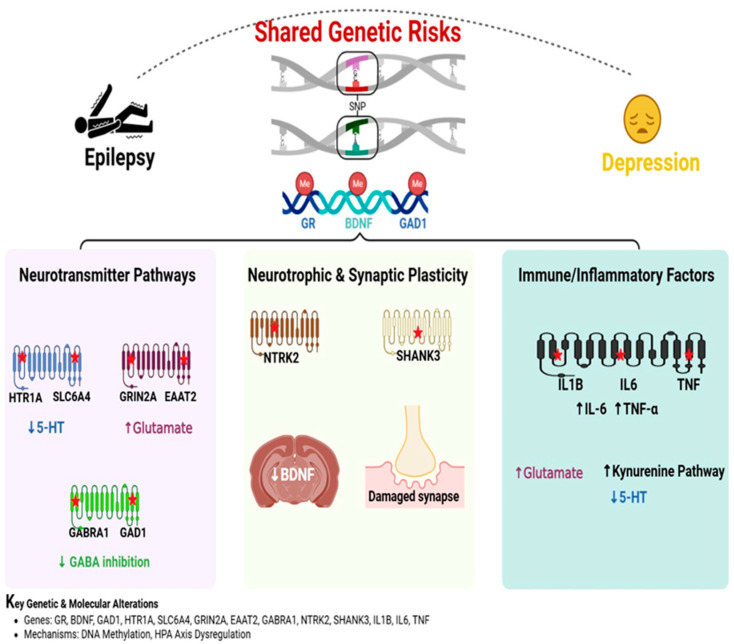
Shared genetic risk factors linking epilepsy and depression.

The strong link between epilepsy and depression is increasingly explained by shared genetic risk factors, with genome-wide analyses identifying common genes and mechanisms for both conditions [90]. Data shows that 30–35% of epilepsy patients develop depression, while individuals with depression have a 2–3 fold higher risk of developing epilepsy—a two-way connection indicating shared genetic factors [91]. Large-scale genetic studies have identified shared risk genes that influence critical brain systems: neurotransmitters like serotonin and glutamate, growth factors such as BDNF, and immune regulators, including IL-6 and TNF-α [92]. Family studies support this by showing higher rates of mood disorders among relatives of epilepsy patients, reinforcing inherited vulnerability. The following discussion details these shared genetic mechanisms [90,93,94] (Figure 3).

Single-nucleotide polymorphisms (SNPs) and epigenetic modifications (methylation) in genes such as glucocorticoid receptors (GRs), brain-derived neurotrophic factor (BDNF), and glutamic acid decarboxylase 1 (GAD1) contribute to susceptibility. Dysregulation encompasses decreased serotonin (5-HT) levels through HTR1A and SLC6A4, increased glutamate via GRIN2A and EAAT2, and reduced GABA inhibition due to alterations in GABRA1 and GAD1. Impaired NTRK2 and SHANK3, along with reduced BDNF levels and damaged synapses, indicate compromised neuroplasticity. Upregulation of IL-1β, IL-6, and TNF-α drives inflammation, leading to increased glutamate and activation of the kynurenine pathway, which further depletes 5-HT.

### 4.2. Neurotransmitter Pathways: Serotonin, Glutamate, and GABA

Genetic abnormalities affecting serotonin and glutamate systems play a key role in why epilepsy and depression often occur together. Deficiencies in serotonin 5-hydroxytryptophan (5-HT), a well-known feature of depression, are linked to variations in the SLC6A4 and HTR1A genes, which animal studies show can also increase seizure risk [95]. Interestingly, low serotonin levels worsen seizure development in epilepsy models, while antidepressant medications like fluoxetine help prevent seizures in some genetically prone animals [96]. Problems with glutamate signaling, involving genes such as GRIN2A and EAAT2, lead to excessive brain excitation that damages neurons in both conditions [97]. For instance, variants in the MTDH/AEG-1 gene (known for migraine links) disrupt glutamate clearance, causing harmful buildup that affects both mood and seizure control [98]. Additionally, mutations in GABA-related genes (GABRA1, GAD1) reduce the brain’s natural calming mechanisms, creating a state of overexcitation common to both disorders [99].

### 4.3. Neurotrophic and Synaptic Plasticity Pathways

The TrkB-BDNF pathway is recognized as a key mechanism in TLE and depression, where decreased levels of BDNF disrupt brain plasticity in both conditions [100]. Genetic changes affecting BDNF hinder the formation of new hippocampal neurons and synaptic efficiency, while mutations in its tropomyosin receptor kinase B (TrkB) receptor, encoded by NTRK2, alter the wiring of emotional circuits, creating a double vulnerability to seizures and mood instability [101,102]. Similar disruptions are observed in the SHANK3 gene, which has been linked to autism-epilepsy overlap, where defects in this synaptic organizer lead to impaired glutamate connectivity and depression [103]. These findings highlight how genetic impairments in brain nourishment and synaptic maintenance can jointly increase the risk of developing both epilepsy and depression [104].

### 4.4. Immune and Inflammatory Genetic Factors

Increased evidence points to the number of immune-related genes, such as IL1B, IL6, and TNF, that connect epilepsy and depression through inflammatory pathways [105]. Genetic polymorphisms in these genes raise levels of inflammatory mediators (IL-6, TNF-α), which damage the brain’s protective barrier, promote excitatory glutamate transmission, and decrease serotonin synthesis by activating the KP [105,106]. This inflammatory chain reaction is further amplified by the IL-1 receptor/TLR4 system, which is triggered when seizures release HMGB, a pattern recognized within animal models showing both brain inflammation and depression-like phenotypes [105,106].

### 4.5. Epigenetic Modifications and Gene-Environment Interactions

Environmental factors like early-life trauma can increase the risk of depression and epilepsy by causing epigenetic modifications that modify gene activity [107]. Traumatic experiences in early life are linked to heightened DNA methylation, a chemical marker that silences genes, in critical genes such as BDNF (key for brain plasticity) and SLC6A4 (modulates serotonin) [107,108]. These epigenetic tags essentially “turn down” these protective genes. Similarly, childhood adversity can lead to hypermethylation of GR genes, which impairs the body’s ability to regulate stress responses normally [109]. Dysregulation of the HPA axis stress system thus predisposes individuals to depression and seizures. Other epigenetic modifications, like changes in the shape of the GAD1 gene (which controls the production of the calming neurotransmitter GABA), also play a role by reducing the brain’s natural inhibitory functions [110]. Collectively, these epigenetic processes explain how environmental stressors can biologically increase the risk for both neurological and mood disorders by repeatedly altering the function of crucial genes in the brain.

## 5. Genome-Wide Association Study (GWAS) Studies on Shared Genetic Risks in Epilepsy and Depression: Focus on BDNF and SLC6A4

### 5.1. Introduction to GWAS in Epilepsy–Depression Comorbidity

Recent genetic research has transformed our knowledge of the biological links between epilepsy and depression. Major genome-wide association studies have found overlapping risk genes related to brain development (e.g., BDNF) and chemical signaling (e.g., SLC6A4) [111,112]. These genome-wide analyses illustrate that both conditions result from complex combinations of many genetic variations, each contributing a small part to disease risk [111,112]. The two-way interaction between depression and epilepsy, both disorders making each other more likely, appears to depend on genes that affect multiple aspects of brain function, such as neuron communication, inflammation, and stress response [111,112].

### 5.2. GWAS Methodology and Challenges in Studying Comorbidity

The GWAS examines millions of genetic variations, single-nucleotide polymorphisms (SNPs), to identify associations with complex diseases like epilepsy and depression [113]. Although these studies face inherent challenges such as diverse symptom presentations, the additive effects of many genes, and environmental influences, large collaborative efforts like the Psychiatric Genomics Consortium and the International League Against Epilepsy have managed to identify strong genetic risk factors [113,114]. By combining data from multiple studies, researchers have confirmed important risk-related genetic variants, such as the BDNF variant rs6265 and the serotonin transporter gene SLC6A4’s serotonin-transporter-linked promoter region (5-HTTLPR) polymorphism, highlighting the power of large-scale genetic approaches [115,116].

### 5.3. BDNF: A Neurotrophic Link Between Epilepsy and Depression

The BDNF gene encodes brain-derived neurotrophic factor, a protein vital for neuronal health, brain adaptability, and mood [117]. The study indicates its crucial role in both epilepsy and depression through multiple mechanisms [117]. Genetically, the Val66Met allele (rs6265) links to smaller hippocampal volume in both diseases and poorer stress response [118]. Large genetic analyses confirm that BDNF variants significantly raise the risk for major depression and TLE [119]. Functionally, excess BDNF in epilepsy promotes seizure activity, while a deficiency of BDNF in depression is linked to synaptic loss and dysfunction of the stress system. Epigenetic studies show that chemical silencing of BDNF (via hypermethylation) is also strongly associated with depression in patients with epilepsy [120]. Overall, these findings position BDNF as a central factor in connecting seizure susceptibility with mood disorders across genetic, functional, and epigenetic levels.

### 5.4. SLC6A4: Serotonin Transporter Dysfunction in Comorbidity

The SLC6A4 gene is involved in regulating serotonin, which is essential for the effectiveness of antidepressants, and it has been linked to depression and epilepsy through various study designs [111]. The 5-HTTLPR polymorphism, especially the short (S) allele, reduces gene function and is associated with a 1.2-fold higher risk of depression, as well as influencing the stress response [121,122]. Large-scale genetic studies consistently confirm SLC6A4 as a significant risk factor for major depression [122,123]. Additionally, epigenetic changes such as decreased DNA methylation of the SLC6A4 promoter in patients with depression and epilepsy may increase serotonin transporter levels, potentially worsening mood symptoms [107,108]. At a functional level, disrupted serotonin signaling due to SLC6A4 abnormalities contributes to both higher susceptibility to seizures and depressive symptoms by impairing emotional brain networks [107,108]. Collectively, these findings illustrate how SLC6A4 dysfunction links epilepsy and depression.

### 5.5. Other GWAS-Identified Shared Risk Loci

Genetic studies have also identified several other important genes beyond BDNF and SLC6A4 that influence both epilepsy and depression. Variations in immune response genes like IL6 and TNF are linked to treatment-resistant epilepsy and depression, indicating common neuroinflammatory mechanisms [13]. Similarly, ion channel genes such as SCN1A (associated with severe epilepsy conditions like Dravet syndrome) and CACNA1C (linked to mood disorders) show effects in both disorders [124]. Additionally, epigenetic regulator genes like DNMT3A and MECP2, which control DNA methylation patterns, are affected in ways that impact both neurological and mood disorders [125]. These findings highlight multiple biological pathways through which genetic factors may increase susceptibility to both depression and epilepsy.

Experimental and clinical examples from various studies providing data on the key effects and possible mechanisms are presented (Table 1 and Table 2).

## 6. The Interplay Between Gut Microbiota and Gut–Brain Axis in Epilepsy and Depression

Our understanding of CNS disorders like epilepsy and depression has been fundamentally transformed by the complex interaction between the gut microbiota and the gut–brain axis, which demonstrates that these conditions are much more than just brain disorders [47]. A growing body of research clarifies how microbial imbalances can significantly affect brain function and behavior in epilepsy and depression, alongside established links between gut dysbiosis, mood and anxiety, and neurodegenerative diseases like autism and multiple sclerosis [177]. This two-way communication system, controlled by immunological, metabolic, endocrine, and neurological pathways, indicates that changes in the gut microbiota can play an important role in the development of various co-morbidities [178].

The ability of the gut microbiota to produce a wide range of neuroactive compounds is a key process [178]. Bacterial fermentation of dietary fibers yields short-chain fatty acids, including butyrate, propionate, and acetate, which are essential. For instance, butyrate can cross the blood–brain barrier and directly influence neuroinflammation, brain energy metabolism, and epigenetic modifications, thereby affecting synaptic plasticity and neuronal health [179]. Conversely, dysbiosis may lead to a decrease in anti-inflammatory bacterial metabolites or an excess of pro-inflammatory ones [180]. The metabolism of tryptophan is also significantly influenced by gut bacteria. Tryptophan may be diverted from serotonin synthesis—a critical neurotransmitter linked to depression—into the kynurenine pathway when microbiota [181]. This diversion can produce neurotoxic metabolites that trigger neuroinflammation and neuronal dysfunction. In addition to lowering the seizure threshold, these changes can exacerbate symptoms of depression [181].

The gut–brain axis is bidirectional, meaning that chronic stress and central nervous system abnormalities can alter the composition and function of the gut microbiota, creating a vicious cycle [182]. Dietary interventions beyond general healthy eating—such as specific fiber intake to boost SCFA production, fecal microbiota transplantation (FMT), and targeted probiotics and prebiotics to restore microbial balance—are being studied as additional or new treatments for depression and epilepsy [183]. Developing comprehensive, personalized treatments for these complex neurological and mental disorders is greatly promising when the gut microbiota is modulated to positively influence the gut–brain axis, although the exact mechanisms are still being explored [184].

### 6.1. Possible Underlying Mechanisms of Gut Dysbiosis in Epilepsy and Depression

There is a clear correlation between depression and epilepsy pathophysiology and changes in gut flora. The interlink between the microbiome profile for depression and epilepsy is theoretically based on current evidence. Indirect findings from other studies may, however, indicate a shared strain of bacteria that is predictive of depression in epilepsy. As noted previously, depression is an IBS co-morbidity. Significantly, IBS predisposes to seizures [185]. People with IBS have elevated intestinal permeability, as do people with depression [186,187], leading to an increase in lipopolysaccharide (LPS) [188] and high pro-inflammatory cytokines [189,190]. Experiments were conducted on how the ketogenic diet (KD) affected the fecal microbiota in children with epilepsy. Whole-metagenomic sequencing at three months of KD treatment showed a relative decrease in the abundance of Bifidobacteria in patients, confirming the importance of the microbiota in seizure susceptibility and the therapeutic anti-seizure effect of KD treatment. Experiments were conducted to investigate how KD affects the fecal microbiota in children with epilepsy. Complete metagenomic sequencing at the third month of KD treatment showed that the relative abundance of Bifidobacteria was significantly reduced in patients, reflecting the involvement of the microbiota in seizure susceptibility and the potential anti-seizure effect of KD treatment [191]. Depression and IBS patients are less responsive to medication and psychotherapy [182]; therefore, stabilizing gut microbiota composition through probiotics and prebiotics appears to be the best approach to treat depression in epilepsy. Research suggests that imbalanced gut microbiota may cause depression, anxiety, and panic attacks [183,184]. Studies showing that germ-free or microbiota-deficient mice experience impaired brain development and display abnormal mental behavior demonstrate a strong link between depression symptoms and gut microbiota [185,186]. It is also shown that the gut microbiome modulates neuroplasticity and myelin plasticity. Therefore, by promoting the microbiota–gut–brain axis and fostering brain and behavioral development, modulating or correcting microbiome disruptions can aid in treating the disorder. Efforts to intervene in the microbiota using probiotics, prebiotics, and fecal microbial transplantation have made the therapeutic effects of microbiome management or therapies focused on the microbiota more significant. Overall, the proposed link between gut microbiota and depression in epilepsy has gained momentum as a potential therapeutic avenue. The gut microbiota plays a vital role in maintaining the host’s mood, brain function, and emotions by utilizing the microbiota–gut–brain axis, which consists of three pathways: the neurological, neuroendocrine, and immunological systems [192]. Regulating neurotransmitter-mediated metabolic networks and even gene transcription associated with neurotransmitters, the gut microbiota can control the production of neurotransmitters. Certain types of bacteria, such as Bacillus and certain LAB forms, produce different neurotransmitters, e.g., catecholamines and acetylcholine [193], whereas 5-HT can be synthesized by *Streptococcus*, *Escherichia*, *Enterococcus*, and *Candida* [194], and this contributes to 90% of the body’s 5-HT [195]. Several coryneform and LAB types also produce GABA and glutamate [196]. Since the etiology of epilepsy and depression is thought to be a failure of neurochemistry, the neuroactive action of gut microbiota modulation could also be further investigated as a cure or treatment for depression in epilepsy.

### 6.2. Therapeutic Basis and Mechanistic Evaluation of Probiotics in Gut–Brain Axis Modulation

**Figure 4:**
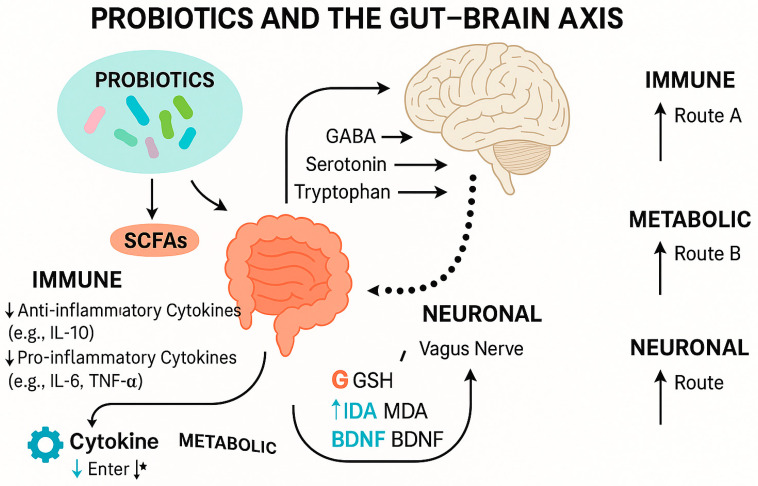
Conceptual illustration of probiotic–gut–brain interactions. * Star symbol: denotes overlapping/multifactorial cytokine actions (immune, metabolic, and neuronal).

Recent evidence from both preclinical and clinical studies has provided a clearer understanding of the mechanistic pathways through which probiotics may influence neurological and psychiatric conditions such as epilepsy and depression. Specific strains, including Lactobacillus rhamnosus, Bifidobacterium longum, *L. plantarum*, and *L. casei*, have demonstrated therapeutic potential by modulating inflammatory and neurochemical biomarkers. Probiotics have been shown to reduce pro-inflammatory cytokines like IL-6, IL-1β, and TNF-α [197,198], while enhancing anti-inflammatory responses such as IL-10. Additionally, *L. rhamnosus* (JB-1) modulated GABA receptor expression in key brain regions via the vagus nerve, leading to anxiolytic and antidepressant effects in animal models [199]. In clinical settings, a randomized controlled trial involving patients with Alzheimer’s disease showed that a multi-strain probiotic increased serum brain-derived neurotrophic factor (BDNF) by 36%, elevated antioxidant enzyme levels (SOD), and reduced oxidative markers like malondialdehyde (MDA) and protein carbonyl content [200]. These findings support the concept that probiotics may modulate the gut–brain axis through mechanisms involving immune signaling, neurotransmitter biosynthesis, and microbial metabolite production (e.g., SCFAs) (Table 3 and Figure 4).

This figure illustrates the interaction between probiotic strains and gut–brain axis pathways, highlighting their roles in modulating neurotransmitters, signaling the immune system, and providing antioxidant defense. Probiotics influence central nervous system function through vagal pathways, cytokine modulation (e.g., IL-1β, IL-6), increased neurotrophic factors (e.g., BDNF), and the production of microbial metabolites, such as short-chain fatty acids (SCFAs). * The star symbol indicates that cytokines can influence gut–brain axis signaling through multiple overlapping pathways, including both direct immune modulation and indirect effects via metabolic and neuronal routes.

### 6.3. Selection Criteria for Neuroactive Probiotic Strains

The probiotic strains highlighted in this review were selected based on mechanistic evidence and demonstrated efficacy in modulating gut–brain axis communication. Several strains, including *L. rhamnosus*, *B. longum*, and *L. plantarum,* have been shown to regulate neuroimmune markers, stress hormones, and neurotrophic factors in both animal and human models. Their effects on pathways such as GABA signaling, tryptophan–serotonin metabolism, and HPA axis regulation support their potential relevance in neurological conditions involving stress, inflammation, and neurotransmitter imbalance [199,200,201].

### 6.4. Mechanistic Insights and Biomarker Modulation

#### 6.4.1. Inflammatory Pathways

Neuroinflammation is a key feature of several neuropsychiatric and neurodegenerative disorders, including epilepsy and major depressive disorder. Probiotic strains such as *Lactobacillus rhamnosus*, *Bifidobacterium longum*, and *Lactobacillus casei* have demonstrated anti-inflammatory effects through multiple immunoregulatory pathways. These strains reduce pro-inflammatory cytokines like interleukin-6 (IL-6), interleukin-1β (IL-1β), and tumor necrosis factor-alpha (TNF-α), while promoting the production of anti-inflammatory cytokines such as interleukin-10 (IL-10) [156,197,198]. In both kainic acid-induced epilepsy models and clinical trials involving patients with Alzheimer’s disease and treatment-resistant depression, probiotic treatment resulted in notable decreases in systemic and central inflammation markers [200]. These results indicate that immunomodulation is a core mechanism in the gut–brain interaction facilitated by probiotics.

#### 6.4.2. Oxidative Stress Regulation

Oxidative stress is implicated in the pathophysiology of both epilepsy and depression, contributing to neuronal damage and mitochondrial dysfunction. Probiotics can modulate redox homeostasis by enhancing the endogenous antioxidant defense system. Several clinical studies have demonstrated that multi-strain probiotic formulations significantly increase antioxidant enzyme levels, such as superoxide dismutase (SOD), catalase (CAT), and glutathione peroxidase (GPx), while simultaneously decreasing lipid peroxidation markers, including malondialdehyde (MDA) and protein carbonyl content (PCC) [200,201]. These antioxidative effects contribute to neuroprotection, synaptic stabilization, and reduced neuronal apoptosis, supporting the use of probiotics as adjuncts in neurological conditions associated with oxidative stress.

#### 6.4.3. Neurotrophic Factor Modulation

Brain-derived neurotrophic factor (BDNF) is a key regulator of synaptic plasticity, learning, and memory. Dysregulation of BDNF has been reported in both epilepsy and depression, and restoring its levels is considered a therapeutic target. Multiple probiotic strains, including *L. plantarum* and *B. bifidum*, as well as multi-strain formulations, have been shown to upregulate BDNF expression in hippocampal and cortical regions, as well as peripherally in serum samples [198,200,203]. The increase in BDNF levels following probiotic supplementation has been associated with improved cognitive function, enhanced neurogenesis, and the reversal of stress-induced synaptic dysfunction in both animal and human studies.

#### 6.4.4. Microbiota-Derived Metabolites: SCFAs and Tryptophan Metabolism

Numerous metabolites produced by the gut microbiota have a significant impact on host physiology, with short-chain fatty acids (SCFAs), such as butyrate, propionate, and acetate, playing crucial roles in gut–brain axis signaling [204,205]. The SCFAs, which are primarily produced by bacteria fermenting dietary fiber, serve as essential energy sources for colonocytes and possess potent immunomodulatory and neuroactive properties [204]. Due to its ability to penetrate the blood–brain barrier and play a neuroprotective role, butyrate, in particular, has garnered attention [204]. Because it inhibits histone deacetylase (HDAC), it controls the expression of genes involved in mitochondrial function, neurotransmitter metabolism, and neural plasticity [204]. Dysbiosis resulting in impaired SCFA production in epilepsy can disrupt the gut barrier, enabling pro-inflammatory bacterial products to get into the systemic circulation and eventually the brain, increasing neuroinflammation and seizure vulnerability [204,206]. Alternatively, the depressive phenotype seen in epileptics may be caused by the inhibition of neurogenesis and synaptic function caused by the lack of protective SCFAs, particularly butyrate [206]. There is a two-way relationship because stress, co-exposure, and repeated seizures can change the composition of the gut microbiota, which in turn affects the production of SCFA. By restoring the integrity of the gut barrier and promoting neuroprotection, SCFA supplementation or microbiota modulation can lessen the frequency of seizures and depressive symptoms [47].

In addition to SCFAs, the gut microbiota has a significant impact on the metabolism of tryptophan, a crucial pathway for the production of serotonin, a neurotransmitter associated with mood regulation. There are two main pathways by which tryptophan is broken down: the kynurenine pathway (KP) and the serotonin pathway [207]. The activity of indoleamine 2,3-dioxygenase (IDO), an enzyme that reroutes tryptophan into the KP, is markedly increased in chronic inflammation, a disease that frequently manifests as epilepsy and depression [207]. Reduced serotonin production and the development of neuroactive kynurenine metabolites, some of which, like quinolinic acid, are excitotoxic and neurotoxic, may arise from this, perhaps causing neuronal damage and heightened vulnerability to seizures [52]. The gut microbiota can directly influence the balance of these metabolites; for example, certain bacteria can produce metabolites of tryptophan that bind to the aryl hydrocarbon receptor, thereby influencing immunological responses and neuroinflammation [181]. Increased IDO activation can be facilitated by dysbiotic guts with an imbalance of particular microbial species. This can lead to a detrimental shift in tryptophan metabolism, which is responsible for the development of seizures and depressive symptoms [181]. Therefore, a possible treatment strategy for the comorbidity is to reverse tryptophan metabolism through therapeutic modification of the gut flora.

#### 6.4.5. Immune Signaling

Bidirectional gut–brain connection relies heavily on immune signaling, which is also mainly to blame for the co-occurrence of epilepsy and depression [208]. Chronic systemic inflammation and neuroinflammation are linked to both conditions, and there is strong evidence that the gut microbiota plays a crucial role in regulating these processes [208]. Increased gut permeability can result from dysbiosis, which involves the disruption of the composition and function of gut microbes [208]. Bacterial compounds, including peptidoglycans and lipopolysaccharides (LPS), can enter the systemic circulation through a breached barrier, activating peripheral immune cells and initiating a chain reaction that produces pro-inflammatory cytokines such as TNF-α, IL-1β, and IL-6 [209]. These circulating inflammatory mediators subsequently either directly penetrate the BBB or activate endothelial cells, which in turn activate the brain’s resident immune cells, namely astrocytes and microglia [209]. Once triggered, these glial cells release additional neuroinflammatory mediators that promote neuronal hyperexcitability and aid in the development of epileptogenesis [209]. At the same time, persistent neuroinflammation impairs neurotrophic factor expression (e.g., BDNF) and neurotransmitter systems (e.g., serotonin, dopamine), leading to synaptic dysfunction and neuronal atrophy, all of which are strongly linked to the pathophysiology of depression [210]. The fact that seizures themselves can cause neuroinflammation, which feeds the vicious cycle of inflammation and exacerbates mood disorders, and that depression and chronic stress can both independently alter the gut barrier and immune responses, perpetuating the vicious cycles of each disease, attests to the reciprocal relationship [210].

Immune signaling has a significant impact on intricate molecular pathways, in addition to its direct cytokine activity. For example, in epilepsy, damaged or stressed cells, such as neurons and glial cells, release the damage-associated molecular pattern (DAMP) HMGB1. TLR4 signaling in immune cells can be activated both centrally and peripherally by HMGB1, resulting in the long-term release of pro-inflammatory cytokines [25]. The gut microbiota can directly affect this system; for example, certain bacterial compounds can directly activate TLR4, thereby increasing the inflammatory response [211,212]. The immune system’s equilibrium can be further tipped in favor of inflammation by dysbiotic gut microbiota, which can also affect the makeup of immune cells by increasing pro-inflammatory Th17 cells and decreasing regulatory T cells. Immunological signaling is a key hub for this gut–brain interaction within this comorbidity, since this systemic and central inflammatory milieu maintains the neurobiological alterations of depression and predisposes to a lowered seizure threshold [213].

#### 6.4.6. Neural Pathways: The Vagus Nerve and Other Neurotransmitters

One of the most important neurological pathways for quick and direct communication between the gut and the brain is the vagus nerve, which also contributes to bidirectional interaction in this comorbidity of depression and epilepsy [214]. Afferent messages from the gut to the central nervous system are mostly transmitted via this long cranial nerve, which reports gut distension, nutrition sensing, and microbial metabolism [214]. By controlling enteroendocrine cells that release neurotransmitters (such as serotonin) or hormones that stimulate vagal fibers, the gut microbiota can either directly or indirectly affect vagal afferents through their metabolites, including SCFAs [204]. Vagus nerve stimulation (VNS), a well-established therapeutic method for refractory epilepsy, is useful in enhancing patients’ mood and quality of life in addition to lowering seizure frequency. It has also been shown that vagal tone is dysregulated in epilepsy. In addition to its anti-inflammatory properties, VNS is believed to have an antidepressant effect through the modulation of noradrenergic and serotonergic processes, highlighting its dual function in both disorders [215].

The bidirectional nature of vagal involvement is crucial. While gut inputs affect brain function through the vagus nerve, central nervous system activity, such as stress and anxiety associated with epilepsy, can also influence vagal efferent activity, which in turn impacts gut motility, secretion, and even the integrity of the gut barrier [216]. Prolonged stress, which is prevalent in people with epilepsy and depression, may cause disturbed vagal tone, which in turn may cause inflammation and gut dysbiosis. These factors then feed back into the brain through afferent vagal pathways, exacerbating neuroinflammation and contributing to mood disorders [217]. The necessity for the vagus nerve to mediate the positive effects of gut microbiota on brain health is highlighted by experimental investigations that demonstrate that loss of vagal integrity can eliminate the antidepressant-like effects of several probiotics [217]. To provide a concrete anatomical and functional basis for the co-evolution and potential therapeutic targeting of depression and epilepsy, the vagus nerve serves as a crucial link between the gut microbiota and the brain’s emotional and epileptic networks [217]. One effective way to reduce seizure activity and depressed symptoms is to modify vagal activity, either directly or indirectly, with probiotics that increase vagal tone or certain dietary therapies [217].

The influence of probiotics on neurotransmitter systems is well established, particularly their role in modulating the levels and activity of GABA (γ-aminobutyric acid), serotonin (5-HT), dopamine, and their precursors. *Lactobacillus rhamnosus* JB-1 has been shown to upregulate GABA receptor expression in cortical and limbic regions via vagus nerve signaling, resulting in anxiolytic effects in murine models [199]. Additionally, Bifidobacterium longum and Lactobacillus plantarum strains have demonstrated the ability to increase tryptophan availability and serotonin biosynthesis in the gut, influencing mood and cognition [218,219]. These neuromodulatory effects have been correlated with improvements in depression and anxiety symptoms in both preclinical and clinical populations.

### 6.5. Distinguishing Probiotics from Live Biotherapeutic Products (LBPs)

While probiotics are commonly used as dietary supplements, LBPs are defined as pharmaceutical-grade live microorganisms intended for therapeutic use in the treatment of diseases. LBPs are subject to rigorous regulatory evaluation for safety, efficacy, and manufacturing standards under agencies such as the FDA and EMA [220]. This distinction is crucial when considering the translational applications of probiotic strains and their potential as targeted biotherapeutics.

## 7. Different Therapeutic Mechanisms

### 7.1. Glutamatergic Modulation: Ketamine and Beyond

Ketamine and its more potent S-enantiomer, esketamine, have emerged as game-changing therapies for patients with both epilepsy and depression, offering a novel approach that hits at the central biological pathways shared by both disorders [221,222]. By modulating glutamate transmission, the brain’s primary excitatory neurotransmitter system is dysregulated in both illnesses. These rapid-acting drugs accomplish something never before achieved, providing simultaneous benefits for seizure control and mood stabilization [221,222,223]. Their therapeutic action begins with NMDA receptor blockade, which initially inhibits hyperactive glutamate transmission but paradoxically triggers a cascade of beneficial effects: transient blocking of inhibitory GABA neurons leads to a regulated burst of glutamate that ultimately activates AMPA receptors and releases BDNF, leading to the formation of new synaptic contacts in the critical brain regions like the hippocampus and prefrontal cortex damaged in both epilepsy and depression [221,222,223]. The ability of the treatment to rapidly reestablish impaired neural circuits is a paradigm shift from symptom treatment to the possibility of changing the course of the disease [221,222]. However, pragmatic constraints are temporary effects (typically in days to weeks) that must be repeated, and the need for close monitoring due to possible dissociative side effects as well as abuse potential [223].

With response rates frequently surpassing 50% and significant remission rates, esketamine, especially when administered intranasally, has shown unparalleled efficacy in treating treatment-resistant depression (TRD) in clinical settings. It outperforms conventional antidepressants and even performs comparably to electroconvulsive therapy in certain situations. Suicidal thoughts and other depression symptoms are quickly alleviated by it. Beyond its short half-lives and potential for addiction, ketamine and esketamine have certain practical problems when used therapeutically. Acute adverse effects that require supervised administration include dose-related dissociation, headache, and nausea, as well as transient elevations in heart rate and blood pressure. Although clinical trials using therapeutic amounts of esketamine reveal cognitive stability or even improvement, long-term administration, particularly at high or abusive levels, is associated with risks of bladder disease and probable cognitive impairment. Suicidal thoughts and behavior risk is also given a boxed warning and requires close monitoring, especially in pediatric patients. The unique advantages of (R)-ketamine, which may have longer-lasting antidepressant and anti-inflammatory effects with fewer psychotomimetic side effects, are being intensively investigated in ongoing research.

### 7.2. AMPA Receptor Potentiators (AMPAkines) for Epilepsy–Depression Comorbidity

Scientists are getting excited about a new class of drugs called AMPAkines (like LT-102) that could help people suffering from both epilepsy and depression, two conditions that often go hand-in-hand [224]. These drugs work by fine-tuning how brain cells communicate through a system called glutamate signaling, which goes haywire in both disorders [224]. Interestingly, they can help fix damaged brain connections in different ways for each condition [225]. For depression, they rebuild lost connections in the brain’s mood control center (the prefrontal cortex) by activating a special growth pathway [225]. For epilepsy, they calm down overactive brain circuits by stabilizing specific receptors [225].

### 7.3. Metabotropic Glutamate Receptor 5 (mGluR5) Antagonists for Epilepsy–Depression Comorbidity

Researchers are increasingly interested in drugs that block a brain receptor called mGluR5 (like methyl-phenyl-ethynyl-pyridine (MPEP) and methyl-thiazolyl ethynyl pyridine (MTEP), which have real potential to treat both epilepsy and depression simultaneously [226,227]. The two diseases often occur together, and these experimental medications seem to treat both conditions at their source by calming abnormally active brain function and quelling inflammation [226,227]. The mGluR5 receptor serves as an emotional volume control for brain centers such as the hippocampus and amygdala. Dialed down with these blockers, it rebalances in many different ways [226,227]. For epilepsy, they will shut off seizures by blocking toxic overactivation of other receptors (NMDA) and suppressing surges of calcium in brain cells [226]. For depression, they re-establish normal relations in mood-depressing areas by repairing glutamate levels and activating BDNF, a brain growth factor of significance [228]. A notable fact is that these drugs target brain inflammation, quieting hyperactive immune cells and releasing toxic chemicals (including IL-6 and TNF-α) that worsen both diseases [228]. They also help the brain break down tryptophan (serotonin precursor) in a less toxic way, producing protective chemicals instead of toxic ones [229]. A few of the clinical trials for similar drugs have been unpredictable, reminding us that the human brain is more complex than rodent models [230].

Although mGluR5 antagonists (e.g., MPEP, MTEP) have a strong preclinical case for treating epilepsy and depression comorbidity, their path to clinical viability for comorbidity is not entirely straightforward [226]. Finding a treatment window when glutamatergic modulation can effectively lower seizure activity and elevate mood without producing undesirable neurological or psychiatric side effects is one of these challenges [226]. Although the goal of these antagonists is to restore normal excitability, severe or protracted mGluR5 blocking might impair critical physiological processes that depend on mGluR5 signaling, including memory, learning, and motor coordination. Furthermore, a worldwide antagonism may not always be desirable due to the variable roles that mGluR5 plays in various brain circuits and regions involved in either depression or epilepsy [231]. Given the complex heterogeneity of human depression and epilepsy with a wide range of aetiologies and comorbidities, it is also likely that response to mGluR5 antagonists would be highly variable. To mitigate the unpredictable outcomes observed in some early clinical trials, strict patient stratification and the development of biomarkers for predicting treatment response would be necessary.

### 7.4. Epigenetic Therapy

#### DNA Methyltransferase (DNMT) Inhibitors for Epilepsy–Depression Comorbidity: Insights from Preclinical Studies

Scientists are studying a potential new approach to curing epilepsy and depression at the same time, to be treated with drugs called DNMT inhibitors (e.g., decitabine and azacytidine) that are molecular erasers of unnecessary chemical marks on DNA [232]. In epilepsy, overactive DNMT enzyme activity represses genes necessary to calm brain signals (e.g., GAD67) and brain cell growth (e.g., BDNF), leading to seizures and mood disturbances [233,234]. However, the good news is that when scientists administer these eraser medicines for DNA to animals with epilepsy, they observe the brain rewriting its instruction manual: the BDNF gene is reactivated, seizures become less frequent, and depressive behaviors improve. Similar drugs like zebularine even repair problems with serotonin transport, which may be responsible for their double whammy.

Besides simply fixing gene function, these drugs also appear to calm brain inflammation—they lower levels of errant immune molecules (IL-6, TNF-α) and enable the brain to metabolize tryptophan (the precursor to serotonin) in healthier ways. They work best when combined with other epigenetic drugs, such as valproate [234]. These drugs can affect the whole body, struggle to reach the brain in sufficient concentrations, and might cause other problems if taken long-term [234,235,236]. Therefore, scientists are developing smarter forms (such as MG98) that target only the pesky enzymes. In contrast, others push the envelope of clustered regularly interspaced short palindromic repeats (CRISPR) technology to edit these DNA labels with pinpoint accuracy [237]. While we’re not yet ready for treatments in humans, this study opens up an entirely new train of thought about how to treat these disorders, not just managing the symptoms, but potentially fixing the underlying genetic misprogramming that causes both epilepsy and depression in vulnerable individuals.

There are particular and important difficulties in converting DNMT inhibitors from encouraging preclinical data to effective clinical treatments for the comorbidity of epilepsy and depression [238]. While the lack of specificity in many of the available DNMT inhibitors is a significant obstacle, epigenetics presents an enticing mechanism for long-term neuronal and glial plasticity [238]. These substances can alter gene expression in unexpected ways throughout the body, not only in specific brain regions or cell types involved in comorbidity but also in DNA methylation throughout the genome, potentially with off-target effects [238]. A challenging pharmaceutical objective is brain penetration combined with systemic safety and lack of side effects, especially when prolonged dosing is needed for depression and epilepsy. Furthermore, it remains unknown how to determine the ideal dosage and duration of epigenetic modification without causing widespread dysregulation. Individualized treatments are particularly challenging, and the complexity and dynamism of the human epigenome also suggest that results obtained in controlled preclinical models may not necessarily translate to the highly diverse genetic and environmental backgrounds of human patients.

### 7.5. Probiotic Interventions for Epilepsy–Depression Comorbidity: Insights from Preclinical Studies

It turns out that the bacteria living in our guts might hold a key to treating both epilepsy and depression, two conditions that often go hand-in-hand [239]. To effectively control epilepsy, probiotic supplements may be suggested as an adjuvant therapy to reduce depressive symptoms. Since melancholy in epilepsy is a diverse condition, altering the gut flora may be just as beneficial as taking medicine. Most antidepressants have physiological effects right away, but the therapeutic impact takes weeks to manifest, and in some patients (15–30%), the adverse effects may cause them to stop taking the medication [240]. The term “encephalobiotics”, which encompasses probiotics, prebiotics, postbiotics, microorganisms, microbial components, or any substance that may alter the microbiota to enhance cognition, has recently been introduced [241,242].

Psychobiotics, on the other hand, are living bacteria (probiotics) that, when consumed by individuals with mental health disorders, interact with the commensal gut bacteria to provide restorative mental benefits [243]. It is possible to remove some obstacles to successful treatment by using these methods to reduce the symptoms of depression in people with epilepsy. Nevertheless, further research is required to fully understand how these medicines work to treat depression in people with epilepsy. One could suggest that the mechanism of action entails controlling neurotransmission and inflammatory indicators [244].

Scientists are discovering that certain probiotics, such as *Bifidobacterium longum*, *Lactobacillus acidophilus*, and *Lactobacillus rhamnosus*, can actually reduce seizures and improve mood in animal studies, likely by calming brain inflammation, balancing neurotransmitters, and resolving communication breakdowns along the gut–brain axis [245,246]. For example, in rats with chemically induced seizures, probiotics didn’t just make seizures less severe, they also improved memory and reduced depressive behaviors, possibly by modulating neuroinflammation by lowering pro-inflammatory cytokines (e.g., IL-6, TNF-α) and enhancing anti-inflammatory signaling, thereby mitigating the chronic inflammatory state common to both disorders, and boosting protective factors like BDNF, a protein crucial for brain health [247]. Also, by shifting tryptophan metabolism away from neurotoxic QUIN toward serotonin synthesis, probiotics address the serotonin deficiency observed in depression and the glutamate excitotoxicity implicated in seizures [248].

One of the most effective ways probiotics work is by producing short-chain fatty acids, particularly butyrate, which acts as a protective barrier for the brain. Butyrate strengthens the blood–brain barrier, reduces toxic glutamate buildup, and even helps the brain make more GABA (a natural calming chemical) [246,249]. It also tweaks how the body processes tryptophan, steering it toward making serotonin instead of neurotoxic byproducts that worsen seizures and mood. Additionally, probiotics may help mitigate some adverse side effects of anti-seizure medications, such as brain fog and low mood, by protecting gut bacteria and reducing leaky gut syndrome [246,249,250].

Beyond the preliminary findings on inflammation, neurotransmission, and SCFA synthesis, preclinical research is gradually elucidating a more complex set of processes by which probiotic treatment improves comorbidity between epilepsy and depression. According to available data, probiotics can help reduce oxidative stress (OS), a pathological factor that contributes to neuronal damage and dysfunction in both disorders [251]. Research shows that different probiotic strains and combinations can lower OS markers like malondialdehyde (MDA) and nitric oxide (NO) while also increasing the body’s natural antioxidant defenses like glutathione (GSH) and total antioxidant capacity (TAS), especially in animal models of epilepsy [252]. Certain probiotic mixtures, for example, have been shown to stimulate beneficial antioxidant enzymes and signaling pathways, such as the nuclear factor erythroid 2-related factor (Nrf2)/Heme oxygenase 1 (HO-1) signaling pathway, which is essential for protecting cells from oxidative damage and inflammation. This leads to neuroprotection and a decreased risk of seizures. Beyond reducing inflammation in the brain, this direct antioxidant effect provides a fundamental defense against the persistent cellular damage common to both conditions [253].

Another component of probiotic action is their profound influence on the HPA axis, the body’s central stress response system. Preclinical evidence demonstrates that some Psychobiotics possess the ability to modulate HPA axis activity, thereby attenuating stress-induced elevations of corticosterone (the rodent equivalent of cortisol) and precluding the negative effects of chronic stress on brain function and gut integrity [254]. Dysregulation of the HPA axis is a chronic feature of both depression and epilepsy, resulting in impaired neuroendocrine homeostasis and increased vulnerability to seizure activity and mood disruption. Probiotic interventions, by restoring HPA axis homeostasis, indirectly contribute to neurogenesis—the formation of new neurons, particularly in the hippocampus, a brain region crucial for mood regulation and memory [254]. Although BDNF is mentioned in passing in the provided text, other preclinical research indicates that some probiotics can increase other neurotrophic factors, such as Nerve Growth Factor (NGF), in specific brain regions. This further supports neuronal survival, plasticity, and resilience to damage from seizures and mood disorders [255]. Probiotics have the pleomorphic potential to directly affect shared pathophysiological processes involved in the complex interplay of depression and epilepsy, going beyond symptomatic management to improve underlying biological vulnerabilities holistically. This is demonstrated by the additive effects of lowering oxidative stress, normalizing the HPA axis, and improving neurotrophic support [256].

There are several practical and scientific challenges in converting some probiotic medicines from encouraging preclinical findings to successful clinical treatments for the comorbidity of epilepsy and depression [257]. The inherent diversity of human gut microbiomes, which are significantly impacted by lifestyle, nutrition, genetics, and drug usage, is one of the challenges. This is due to the possibility that a probiotic strain or consortium that works well in a regulated animal model might not colonize or have the same beneficial effects in diverse human populations. Maintaining the viability, stability, and controlled delivery of live bacteria to the gut in therapeutic doses is technically challenging, making dosage and formulation essential considerations [258]. Formulation optimization is also problematic, as it is challenging to identify the precise strain-specific processes and metabolites that provide therapeutic effects on the gut–brain axis. Clinical development is made more difficult by the fact that regulatory channels for “live biotherapeutic products” are still being developed. As we go from generalized preclinical findings to precision microbiome-based medicine, the ultimate success of probiotic therapy for comorbidity still depends on rigorous human studies to determine efficacy, safety, the optimal dosage, and which patient subsets are most likely to respond [258].

The relationship between the intestinal and the CNS should be studied since they might cooperate to create the positive effects of biotic supplements. Before these treatments can be considered a first-line treatment for reducing the symptoms of depression in epilepsy, more research is required, including more thorough investigations on both humans and animals [259].

### 7.6. Future Treatments

Future treatment choices for epilepsy and depression comorbidity will unavoidably include synergistic combinations of traditional medicines with novel approaches that directly target the gut–brain axis, in addition to the present therapeutic modalities [260]. Because it acknowledges the intricate interconnectedness of neurological and psychiatric processes coordinated by gut-derived signals, this hybrid approach is especially promising. One of the main approaches combines well-established antidepressants and anti-epileptic medications (AEDs) with interventions that target the microbiota. Although the main mechanisms of action of AEDs and antidepressants are brain circuits and neurotransmitter systems, side effects and non-response rates might undermine their therapeutic efficacy [261]. Combining strategies such as FMT, customized probiotic and prebiotic supplements, or even engineered commensal bacteria may enhance treatment. For example, AEDs and probiotics containing bacteria that produce SCFA, like Lactobacillus species and Bifidobacteria, can be taken together. In addition to strengthening gut barrier function and reducing systemic inflammation, the SCFAs generated would also directly affect neurotransmitter production and neuroinflammation in the brain, lowering seizure thresholds and elevating mood beyond what AEDs alone can provide [262]. The goal of this synergistic treatment is to create an ideal gut environment that supports a healthy brain, making the brain more receptive to traditional pharmaceutical agents and possibly lowering the need for dosage or adverse effects.

Moreover, the combination of conventional therapies with novel anti-inflammatory strategies is another priority for the future. Because of the general implication of neuroinflammation in epileptogenesis and depression pathophysiology, overt reduction in inflammatory mechanisms can be profoundly therapeutic [263]. This could involve novel anti-inflammatory drugs targeting major mediators, such as IL-1β, TNF-α, or HMGB1, selectively, given in combination with AEDs [212,263]. Most importantly, these anti-inflammatory drugs can be used in conjunction with gut-modulating treatments to achieve a dual anti-inflammatory effect, preventing both central neuroinflammation and systemic inflammation originating in the gut that projects back into the brain [263]. For instance, centrally active anti-inflammatory drugs could be used in conjunction with microbiota-targeting treatments that reduce the relative presence of pro-inflammatory bacteria or increase the synthesis of anti-inflammatory metabolites (such as certain SCFAs) [263]. By reducing inflammation in the central and systemic milieu, this synergistic regulation would decrease neuronal hyperexcitability and prevent the typical conditions of synaptic dysfunction and neuronal death. Additional research into therapies that target the kynurenine pathway specifically to change tryptophan metabolism from neurotoxic to neuroprotective metabolites, possibly using enzyme modulators or targeted microbial interventions, could lead to additional therapeutic benefits [264]. To restore homeostasis of the gut–brain axis and provide these individuals with more effective and long-lasting relief, a balanced, personalized medical approach that combines dietary, microbial, immunological, and medication-based therapies is what the future holds.

## 8. Limitations and Future Perspectives

Despite massive advances towards understanding the comorbidity between epilepsy and depression, some constraints are holding back progress. Studies in existence are typically plagued with clinically heterogeneous populations, with studies predominantly focused on severe epilepsy (e.g., drug-refractory epilepsy) or some subtypes (e.g., TLE), limiting generalizability. In addition, while preclinical models demonstrate potential therapeutic mechanisms, such as glutamatergic modulation (ketamine, AMPA antagonists), anti-inflammatory strategies (IL-1β blockade), and epigenetic drugs (DNMT inhibitors), preclinical observations have still been challenging to translate to humans due to differences in disease complexity and drug tolerability. The majority of new medications, including mGluR5 antagonists and probiotics, demonstrate efficacy in animal models but exhibit variable effectiveness in humans, underscoring the need for more accurate biomarkers to stratify patients and predict response. Further, the reciprocally interactive relationship between epilepsy and depression also makes it difficult to treat because the conventional treatments have largely addressed single symptoms rather than the shared pathways, like neuroinflammation, HPA axis dysregulation, or synaptic dysfunction. The transient benefits of rapid-acting medications (e.g., ketamine) and systemic side effects of epigenetic therapy point to the necessity for more enduring and brain-specific treatments.

There are still many obstacles to overcome before these promising preclinical findings, which point to new targets like DNMT inhibitors, mGluR5 antagonists, and specific probiotic species, can be translated into safe and effective human treatments for the comorbidity of epilepsy and depression. The intricacy of the human gut–brain axis, which is significantly more complex and dynamic than in animals, is one such significant obstacle. Human genetics, lifestyle, nutrition, and continuous prescription regimes can all significantly alter the gut microbiota’s composition and function, making it impossible to reliably test the safety and effectiveness of microbiota-modifying treatments. Furthermore, it remains challenging to utilize DNMT inhibitors and mGluR5 antagonists without inducing off-target side effects. These medications have a pleiotropic effect, and significant pharmacological optimization is needed to achieve selective efficacy on specific neural circuits or cell types of interest in both epilepsy and depression, with a low risk of adverse effects. Since a medication that works well for one will unpredictably aggravate the other or result in unexpected interactions, comorbidity type is also troublesome. As a result, thorough clinical studies are necessary to close the gap in translational research, which encompasses not only the effectiveness of individual medications but also their safety profiles and potential synergies of combination regimens in various patient populations.

Future research must emphasize precision medicine strategies based on genetic (e.g., BDNF, SLC6A4 polymorphisms) and inflammatory biomarkers (e.g., HMGB1, IL-6) to guide tailor-made treatments. New technologies, such as CRISPR-based epigenetic modification and gut microbiome modulation, hold promise for reversing molecular dysregulation with reduced off-target effects. Combinations of treatments, e.g., ketamine combined with anti-inflammatory drugs or probiotics combined with neuromodulation, may enhance both efficacy and long-term maintenance. Clinically, neuropsychiatry convergence using multidisciplinary models of care can enhance early detection and management of depression in epilepsy patients, especially in at-risk populations (e.g., children with early life stress, older patients with neurodegeneration). Long-term studies are essential to investigate how early interventions (e.g., stress management, anti-inflammatory diets) could prevent the development of comorbidity. Ultimately, bridging the gap between preclinical discovery and clinical translation will require collaborative efforts, innovative trial design, and a shift in paradigm toward targeting therapies to shared biological mechanisms rather than discrete symptoms. Addressing these challenges, therapeutics in the future can not only alleviate symptoms but also change the disease trajectory, holding promise for improved quality of life for affected individuals.

## 9. Conclusions

The intricate interaction between epilepsy and depression is a result of shared etiologies in neuroinflammation, synaptic dysregulation, and derangement in stress systems, creating a self-perpetuating cycle where each illness feeds into the other. Neuroinflammatory conditions, when activated over a prolonged period by cytokines such as IL-6 and TNF-α, not only lower seizure thresholds but also disrupt mood-regulating networks. HPA axis hyperactivity, driven by hippocampal damage and cortisol toxicity, further entrenches this comorbidity. Genetic and epigenetic studies reveal how BDNF, SLC6A4, and immune disease-associated gene variants predispose to both conditions, with early trauma causing enduring molecular damage by DNA methylation. This overlap of mechanisms is why treatments with standard medicines are often unsuccessful: they target symptoms in isolation without addressing the underlying shared biology. However, new therapies are in development. The antidepressant and potential antiseizure actions of ketamine, which are demonstrated within minutes, illustrate how glutamate modulation can restore synaptic connectivity. Meanwhile, BDNF and mGluR5 antagonists modulate excitatory signaling with precision.

Epigenetic therapies, such as DNMT inhibitors, are promising for reversing maladaptive gene silencing, and probiotics suggest the gut microbiome’s role in suppressing inflammation and promoting neuroprotection. Yet, challenges remain; scaling preclinical efficacy into the clinic requires getting around hurdles such as treatment longevity, individualized dosing, and minimizing side effects. The future is combinatorial: combining ketamine with anti-inflammatory agents, utilizing CRISPR to edit epigenetic marks, or pairing probiotics with neuromodulation. Most importantly, this changing comprehension requires the deconstruction of the old dichotomies between neurology and psychiatry. With the recognition that epilepsy and depression are manifestations of the same interrupted networks, clinicians may embrace interdisciplinary models of care that treat the two as interdependent conditions.

For patients, this paradigm offers more than just symptomatic treatment; it holds the promise of therapies targeting etiologic roots, enhancing quality of life, and potentially modifying disease pathways. The future of treatment for this comorbidity will depend on ongoing research into biomarkers, innovative therapeutics, and team-based care models, ultimately transforming our approach to these interconnected diseases.

## Figures and Tables

**Table 1:** Different preclinical models of epilepsy and its comorbidity (depression).

Preclinical Studies on Epilepsy–Depression Comorbidity				
Experimental Model	Key Effects	Proposed Mechanisms	References	Limitations
Pentylenetetrazol-kindled rats	↑ Depressive-like behavior in forced swim test (FST), ↓ hippocampal BDNF	GABAergic dysfunction, neuroinflammation (↑ IL-6, TNF-α)	[126]	Limited to animal models, specific tests, and human translation is difficult.
Pilocarpine-induced TLE (rats)	↑ Anxiety/depression in (elevated plus maze, FST), hippocampal atrophy	Limbic hyperexcitability, HPA axis dysregulation (↑ cortisol)	[127,128,129,130]	Limited to animal models, specific tests, and direct human translation is difficult.
Kainic acid-induced status epilepticus (rats)	Persistent depressive phenotypes, synaptic loss in the prefrontal cortex	Glutamate excitotoxicity, ↓ neurogenesis	[131,132]	Limited to animal models, specific findings, and human translation is difficult.
Genetic epilepsy (genetic absence epilepsy rats)	Comorbid absence seizures + depression-like behavior	Thalamocortical dysrhythmia (T-type Ca^2+^ channels), serotonin deficiency	[133,134]	Limited to genetic animal model, specific findings, and human translation.
Chronic stress + TLE (mice)	Exacerbated seizure frequency + anhedonia (sucrose preference test)	Neurosteroid withdrawal (↓ allopregnanolone), ↑ CRH in the amygdala	[135,136]	Limited to animal model, specific findings, human translation difficult.
Flinders Sensitive Line (rats)	Spontaneous seizures + despair behavior (FST)	Serotonin transporter (SLC6A4) dysfunction, ↑ KP activity	[137]	Limited to animal models, specific findings, and human translation is difficult.
WAG/Rij rats (absence epilepsy)	Spike-wave discharges + depressive-like immobility	T-type Ca^2+^ channel hyperactivity, ↓ noradrenaline in locus coeruleus	[138,139]	Limited to animal models, specific findings, and human translation is difficult.
Corticosterone-treated mice	↑ Seizure susceptibility + despair (FST)	GR receptor resistance, ↓ hippocampal neurogenesis	[129]	Limited to animal models, specific findings, and human translation is difficult.
Post-status epilepticus depression model (rats)	Spontaneous seizures + social withdrawal	Neuroinflammation (microglial activation, ↑ IL-1β), ↓ mammalian target of rapamycin signaling	[127,140]	Limited to the rat model of post-status epilepticus, specific behavioral and mechanistic findings, direct translation to human comorbidity is difficult.
Dravet syndrome (Scn1a+/− mice)	Severe seizures + anxiety/depression	Nav1.1 channel dysfunction, 5-HT depletion	[141,142]	Limited to the genetic animal model of Dravet syndrome, specific findings, and direct human translation are challenging.
Early-life stress + TLE (rats)	↑ Epileptogenesis + adult depressive behavior	Epigenetic modifications (BDNF methylation), HPA axis hyperactivity	[143,144,145]	Limited to animal models of early-life stress and TLE, specific findings, and direct human translation are challenging.
Ketogenic diet (TLE mice)	↓ Seizures + improved mood (FST)	↑ GABA/glutamate ratio, ↓ neuroinflammation (IL-6, HMGB1)	[146,147]	Small sample size, short duration, unclear long-term effects, and species-specific responses.
IL-1β-infused epilepsy (rats)	Resistant to selective serotonin reuptake inhibitors (SSRIs), persistent depression	KP shift (↑ QUIN), ↓ serotonin synthesis	[128,148]	Single model, acute IL-1β effects, lacks human translation, limited mechanistic depth.
Vagus nerve stimulation (TLE rats)	Reduced seizures + antidepressant effects	↑ Noradrenaline/serotonin in limbic regions, BDNF restoration	[149]	Species-specific, invasive, unclear long-term effects, and lacks human clinical correlation.
Fenfluramine-treated (Down syndrome mice)	↓ Seizures + improved social interaction	Dual 5-HT/sigma-1 receptor modulation, ↑ GABAergic inhibition	[150]	Mouse model only, short-term effects, lacks human data, potential side effects.

↓; Decrease, ↑; Increase.

**Table 2:** Different clinical studies of epilepsy and its comorbidity (depression).

Clinical Studies on Epilepsy–Depression Comorbidity				
Study Population	Key Findings	Proposed Mechanisms	References	Limitations
Newly diagnosed epilepsy patients	A history of depression is 7× more frequent before epilepsy onset	Shared neurobiological vulnerability (e.g., serotonin dysfunction, HPA axis dysregulation)	[151]	Limited scope, new patients, pre-existing depression focus.
Adults with TLE (positron emission tomography study)	Reduced 5-HT_1A_ receptor binding in ipsilateral hippocampus/raphe	Serotonergic deficits in limbic networks	[152]	Limited to TLE adults, specific receptor.
Elderly epilepsy patients (≥55 yrs)	Depression is 3.7× more likely to precede epilepsy	Chronic stress-induced hyperexcitability and hippocampal atrophy	[153,154]	Limited to elderly patients, a specific temporal link.
Patients with drug-resistant epilepsy	A 55% prevalence of depression correlates with seizure frequency	Neuroinflammation (↑ IL-6, TNF-α), BDNF downregulation	[155,156]	Limited to drug-resistant epilepsy, correlational, specific biomarkers.
TLE patients with hippocampal sclerosis	Higher depression rates vs. non-TLE epilepsy	Limbic circuit disruption (amygdala-hippocampus-prefrontal connectivity loss)	[157]	Specific to TLE-HS, focused on limbic circuit disruption.
Pediatric epilepsy cohort	13% depression prevalence; linked to low intelligence quotient/seizure severity	Early-life stress impacts neurodevelopment (e.g., GABA/glutamate imbalance)	[158]	Limited to pediatric cohort, correlational, specific factors, and early stress.
Post-stroke epilepsy patients	No correlation between lesion laterality and depression	Network dysfunction beyond structural damage	[159,160]	Limited to post-stroke, no lesion correlation, network dysfunction focus.
Post-traumatic stress disorder (PTSD) patients with epilepsy	78% reported trauma exposure; 26% met PTSD criteria	CRH overactivation and HPA axis hyperactivity	[161,162]	Limited to PTSD-epilepsy, self-reported trauma, and HPA axis focus.
SSRI-treated epilepsy patients	Sertraline reduced seizure frequency in 60% of cases	Enhanced serotonergic transmission lowers seizure threshold	[163,164]	Limited to SSRI-treated, partial efficacy, and complex mechanisms.
Vagus nerve stimulation-treated refractory epilepsy	Improved mood scores alongside seizure reduction	Noradrenergic/serotonergic modulation via locus coeruleus activation	[165,166]	Limited to VNS-treated, correlational, specific mechanisms.
Epilepsy patients post- electroconvulsive therapy	Rapid antidepressant effects, but memory side effects	Seizure-induced neuroplasticity (BDNF upregulation, glutamate normalization)	[167,168]	
Genetic epilepsy (SCN1A mutations)	Higher depression scores in mutation carriers	GABAergic interneuron impairment	[169,170]	Limited to SCN1A genetic epilepsy, correlational, and specific mechanisms.
Ketogenic diet responders	40% improvement in mood and seizure control	Anti-inflammatory effects (↓ IL-6), enhanced GABAergic tone	[171,172]	Limited to diet responders, specific mechanisms, not all patients.
Epilepsy surgery candidates	Pre-surgical depression predicted worse post-surgical outcomes	Limbic network instability persists despite seizure freedom	[173,174]	Limited to surgery candidates, predictive, network instability focus.
Women with catamenial epilepsy	Higher depression rates linked to hormonal fluctuations	Progesterone withdrawal → reduced allopregnanolone	[175,176]	Limited to catamenial epilepsy, hormonal link, and specific mechanisms.

↓; Decrease, ↑; Increase.

**Table 3:** Summary of Probiotic Strains, Mechanisms, and Neurobiological Effects.

Probiotic Strain	Mechanism of Action	Neurobiological/ Clinical Effect
*Lactobacillus rhamnosus JB-1*	Modulates GABA receptors, vagus nerve signaling.	Anxiolytic, antidepressant effects [199].
*Bifidobacterium longum*	Reduces IL-6, increases BDNF.	Cognitive enhancement, anti-inflammatory [200].
*Lactobacillus plantarum*	Modulates tryptophan–serotonin pathway.	Mood regulation, synaptic plasticity [201].
Multi-strain mix	↑ SOD, ↓ MDA, ↓ IL-1β.	Improved oxidative balance and neuroprotection [198,202].

↓; Decrease, ↑; Increase.

## Data Availability

No new data were created or analyzed in this study. Data sharing is not applicable to this article.

## Abbreviations

The following abbreviations are used in this manuscript:

3-HK	3-hydroxykynurenine
5-HT	5-Hydroxytryptophan
5-HTTLPR	Serotonin-transporter-linked promoter region
AMPA	α-Amino-3-hydroxy-5-methyl-4-isoxazolepropionic acid
AMPAkines	AMPA Receptor Potentiators
BBB	Blood–brain barrier
BDNF	Brain-derived neurotrophic factor
CNS	Central nervous system
CRH	Corticotropin-releasing hormone
CRHR1	Corticotropin-releasing hormone receptor 1
CRISPR	Clustered regularly interspaced short palindromic repeats
DNA	Deoxyribonucleic acid
DNMT	DNA methyltransferase
FST	Forced swim test
GABA	Gamma-aminobutyric acid
GAD	Glutamic acid decarboxylase
GRs	Glucocorticoid receptor
GWAS	Genome-wide association study
HMGB1	High mobility group box 1
HPA	Hypothalamic–pituitary–adrenal axis
IBS	Irritable bowel syndrome
IDO	Indoleamine 2,3-dioxygenase
IL-1R1	Interleukin 1 receptor, type I
IL-1β	Interleukin-1 beta
IL-6	Interleukin 6
KD	Ketogenic diet
KMO	Kynurenine 3-monooxygenase
KP	Kynurenine pathway
KYNA	Kynurenic acid
mGluR5	Metabotropic glutamate receptor 5
MPEP	Methyl-phenyl-ethynyl pyridine
MRs	Mineralocorticoid receptors
MTEP	Methyl-thiazolyl-ethynyl-pyridine
NF-κB	Nuclear factor kappa-light-chain-enhancer of activated B
NMDA	N-methyl-D-aspartate
PTSD	Post-traumatic stress disorder
PVN	Paraventricular nucleus
RAGE	Advanced Glycation End Products
ROS	Reactive oxygen species
SNPs	Single-nucleotide polymorphisms
SSRIs	Selective serotonin reuptake inhibitors
TLE	Temporal lobe epilepsy
TLR4	Toll-like receptor 4
TNF-α	Tumor necrosis factor alpha
TrkB	Tropomyosin receptor kinase B
